# Critical Comparison of Analytical Performances of Two Immunoassay Methods for Rapid Detection of Aflatoxin M_1_ in Milk

**DOI:** 10.3390/toxins12040270

**Published:** 2020-04-22

**Authors:** Ivan Pecorelli, Natascia Guarducci, Cristoph von Holst, Rita Bibi, Michelangelo Pascale, Biancamaria Ciasca, Antonio F. Logrieco, Veronica M. T. Lattanzio

**Affiliations:** 1 Istituto Zooprofilattico Sperimentale dell’Umbria e delle Marche “Togo Rosati”, Via Salvemini 1, 06126 Perugia, Italy; i.pecorelli@izsum.it (I.P.); r.bibi@izsum.it (R.B.); 2 Institute of Sciences of Food Production, National Research Council of Italy, via Amendola 122/O, 70126 Bari, Italy; natascia.guarducci@gmail.com (N.G.); michelangelo.pascale@ispa.cnr.it (M.P.); biancamaria.ciasca@ispa.cnr.it (B.C.); antonio.logrieco@ispa.cnr.it (A.F.L.); 3 European Commission, Joint Research Centre (JRC), Retieseweg 111, 2440 Geel, Belgium; christoph.von-holst@ec.europa.eu

**Keywords:** Aflatoxin M_1_, milk, strip test immunoassay, ELISA, method validation

## Abstract

Aflatoxin B_1_ (AFB_1_) is a secondary metabolite produced by some *Aspergillus spp*. fungi affecting many crops and feed materials. Aflatoxin M_1_ (AFM_1_), the 4-hydroxylated metabolite of AFB_1,_ is the main AFB_1_-related compound present in milk, and it is categorized by the International Agency for Research on Cancer (IARC) as a “group 1 human carcinogen”. The aim of this work was to evaluate and compare the analytical performances of two commercial immunoassays widely applied for the detection of AFM_1_ in milk, namely strip test immunoassay and enzyme linked immunosorbent assay (ELISA). Assay validation included samples at AFM_1_ levels of 25, 50, 75 ng/kg and blank samples (AFM_1_ < 0.5 ng/kg). With respect to a screening target concentration (STC) of 50 ng/kg the two assays showed cut-off values of 37.7 ng/kg and 47.5 ng/kg for strip test and ELISA, respectively, a false suspect rate for blanks <0.1% (for both assays) and a false negative rate for samples containing AFM_1_ at levels higher than STC, of 0.4% (for both assays). The intermediate precision (RSD_ip_) was <32% for the strip test and <15% for the ELISA. Method verification through long-term intra-laboratory quality control (QC) measurements confirmed the results from the validation study. Furthermore, a satisfactory correlation of the results obtained with both immunoassays and the AOAC Official Method 2000.08 was obtained for the analysis of cow milk samples naturally contaminated with AFM_1_ at levels within “not detected” (< 0.5 ng/kg) and 50 ng/kg. Finally, the extension of the scope of the strip test method to goat and sheep milk was evaluated by applying the experimental design foreseen in the EU regulation.

## 1. Introduction

Aflatoxins are mycotoxins found in four main chemical structures: aflatoxin B_1_ (AFB_1_), B_2_ (AFB_2_), G_1_ (AFG_1_) and G_2_ (AFG_2_); they can occur in a wide range of crops, including the major staple cereals (e.g., maize), edible nuts and legumes and their products. The main fungal producers of aflatoxins are *Aspergillus flavus* which produces mainly AFB_1_, AFB_2_ and *Aspergillus parasiticus*, which produces all four forms. Contamination can occur before or after harvest or both. In general, AFB_1_ occurs at the highest levels compared to the others, and is the most toxic and a potent carcinogen [1,2]. AFB_1_ is converted into its hydroxylated metabolite (AFM_1_) by the liver enzymes of lactating animals [3]. This toxin, like the parent compound, has been categorized by the International Agency for Research on Cancer (IARC) as a group 1 toxin, a human carcinogen [2]. Due to their carcinogenity, the aflatoxins uptake through contaminated food consumption should be as low as possible, therefore the aflatoxin legislation is intended to implement the ALARA principle (As Low As Reasonably Achievable) and no threshold limit concerning the tolerable daily intake in humans has been established [4].

Evidences of aflatoxins carry over in milk, edible animal tissues and eggs have been reported, however, among foods of animal origin, milk represents the main source of human exposure to AFM_1_, which is the only mycotoxin which has regulatory limits in milk [4,5,6]. There is evidence of AFM_1_ occurrence in cow milk, but also in milk produced by other ruminants, such as buffalo, goat, sheep and camel [5]. The occurrence of AFM_1_ has been reported in various locations worldwide. Overall, the incidence of AFM_1_ in milk samples and milk products is relatively low in European countries, whereas data from Asian countries like China, Thailand and Taiwan show AFM_1_ occurrence in up to 100% of samples [7]. AFM_1_ is heat stable and processing or storage conditions are ineffective in reducing its concentration in milk and milk products [8,9,10]. Several factors may affect the AFM_1_ contamination of milk, such as environmental conditions, different farming and feeding practices, as well as the quality and safety control systems put in place by food/feed business operators (FBO) [9,11]. The presence of AFM_1_ in milk can be therefore considered as an indicator of maize chain vulnerability to fungal contamination [12]. 

Nowadays, there is an increasing concern for the impact of climate changes (temperature, humidity, rainfall and carbon dioxide production) on fungal behavior and consequently on aflatoxins production [11]. The application of predictive models has already given an indication of the potential increasing contamination by aflatoxins in Europe as consequence of climate changes [13]. Furthermore, a recent study using a full chain modeling approach to predict the impacts of climate change on AFB_1_ production in maize and its consequences on AFM_1_ contamination in dairy cow’s milk, showed that, in the investigated scenario (i.e., Ukrainian maize), AFM_1_ contamination in milk is expected to be comparable or to increase in future climate scenarios [14]. Therefore, according to EFSA definition, the presence of AFM_1_ in milk may be considered as an “emerging risk”, being a known risk for which an increasing and unpredictable pattern of exposure risk is foreseen [15].

Approximately 60 countries have already established regulatory limits for AFM_1_ in milk and dairy products [16]. In the EU, the maximum permitted levels for AFM_1_ have been set for consumable milk (50 ng/kg) [17]. In addition, an alert threshold level of 40 ng/kg calling for action is considered in some EU member states [18]. A maximum permitted level of 500 ng/kg of AFM_1_ in milk has been established by the US-FDA (United States Food and Drug Administration) [19] and by the Codex Alimentarius [20]. This is also the harmonized MERCOSUR limit applied in Latin America [16,21] and in several Asian countries [16].

With the publication of the General Food Law (GFL) [22] the European Union has made a new legal framework laying down the principles, obligations and definitions that apply in the field of food safety. A general principle of the GFL is that FBOs have the primary responsibility for food and feed safety. To this purpose, FBOs must implement a food safety management system, based on the hazard analysis and critical control point (HACCP) principles. Regulatory limits therefore have a strong impact on contracts and procedural guidelines in the dairy industry and, as consequence, on the number of controls needed to verify milk compliance with maximum permitted levels, which may affect production costs.

A wide range of methods for the detection of AFM_1_ in milk and dairy products is currently available, however, achieving key analytical performances, such as sensitivity, precision and reliability, suitable to enforce regulatory limits in the low ng/kg range, is still quite challenging [23]. Screening tests can play an important role within the safety monitoring, allowing rapid decision making and interventions, also affecting the final price of food products. Nowadays, screening tests based on immunochromatographic assays such as dipstick or lateral flow devices, and enzyme linked immunosorbent assays (ELISA) represent the most common formats in the market [24,25,26]. To support FBOs in selecting the most appropriate test in relation to the intended scope, internationally recognized guidelines for screening test performance verification have been made available for instance by the AOAC Research Institute (Performance Tested Methods^SM^) and USDA-GIPSA (Performance Verified Rapid Test), whereas at European level, such guidelines are set in the Commission Decision 657/2002/EC [27] and in the Commission Regulation 2014/519/UE [28], which is specifically devoted to mycotoxin screening methods.

The aim of this work was to evaluate and compare the analytical performances of two commercial immunoassays (strip test immunoassay and ELISA) widely applied for the detection of AFM_1_ in milk. For this purpose, the Commission Regulation 2014/519/UE [28] was taken into consideration as guidance document. Analytical performances, such as precision profile, cut-off value, false positive and false negative rates were evaluated for each assay by single laboratory validation, whereas a verification of the results from the validation study was performed based on long-term intra-laboratory quality control (QC) data. Correlation of the results obtained with the rapid immunoassays and the AOAC Official Method 2000.08 was evaluated for the analysis of naturally contaminated cow milk samples. Finally, the extension of the scope of the strip test method to goat and sheep milk was evaluated by applying the experimental design foreseen in the EU regulation [28].

## 2. Results and Discussion

The experimental design to evaluate analytical performances of strip test immunoassay and ELISA comprised the following steps, which were carried out in parallel for the two assays: i) single laboratory validation study to evaluate precision, cut-off value, false suspect and false negative rates (milk samples fortified by AFM_1_ were used at this stage); ii) verification of cut-off and precision values by long-term intra-laboratory QC study (a QC cow milk sample spiked at 50 ng/kg was used at this stage); iii) evaluation of results correlation between rapid immunoassays and AOAC Official Method 2000.08 (a set of naturally contaminated cow milk samples was used for this purpose). Data obtained for each step are described and discussed in the following.

### 2.1. Validation Results

Validation experiments were performed according to the experimental design described in Section 4.6. The screening target concentration (STC) value was 50 ng/kg. Other tested mass fractions values were: blank (AFM_1_ ≤ 0.5 ng/kg), 25 ng/kg (50% of the STC), 75 ng/kg (150% STC). The same sample set was analyzed by the ELISA and the strip test. Results obtained from the 24 measurements performed for each validation level were taken as basis for the calculation of validation parameters: precision, cut-off value, false positive and false negative rate. The overall results of the statistical assessment are shown in Table 1.

First, precision data were calculated for all tested concentrations. Specifically, RSD_ip_ (intermediate precision) values of 32% (strip test) and 5% (ELISA) were obtained for samples contaminated at 25 ng/kg, values of 17% (strip test) and 10% (ELISA) at 50 ng/kg, 19% (strip test) and 15% (ELISA) at 75 ng/kg. Repeatability values (RSD_r_) were lower than 26% in all cases. Comparable values were obtained for the two tests at STC and above STC, whereas at 50% STC (25 ng/kg) lower intermediate precision values where obtained for ELISA. This could be partially explained by the fact that ELISA was working at a level five times higher than its limit of detection (LOD, 5 ng/kg, see Section 4.4), whereas the strip test was working at its LOD (25 ng/kg see Section 4.3).

With respect to the blank samples, a high relative standard deviation of the strip test response was observed. This could be mainly explained by the fact that for the strip test assay analytical signal values below a certain fixed limit, which is set by the manufacturer, are reported as “zero concentration”, whether that is true or not. This led to a high number of “zero concentration” values in blank samples generating a high standard deviation. However, in the following, it will be shown that, notwithstanding this high value, an acceptable low rate of false suspect results for the blank samples was obtained anyway, due to the good separation of test responses for blank and contaminated samples. 

Overall, the obtained precision values indicated an acceptable robustness of the two test methods, also taking into consideration the very low target levels of AFM_1_ considered for validation.

Once intermediate precision data were available, it was possible to calculate the cut-off values. According to European legislation [28], this value is defined as the response (AFM_1_ mass fraction) obtained with the screening method, “above which the sample is classified as suspect”, with a false negative rate of 5%. The calculated cut-off values were 37.7 ng/kg for the strip test and 47.5 ng/kg for the ELISA test, respectively. In both cases, the assay sensitivity was considered satisfactory for assessing milk contamination at levels encompassing the EU maximum limits.

Based on the cut-off values, the rate of false suspect results was estimated for samples containing AFM_1_ below the STC. Specifically, for samples contaminated at 50% STC (25 ng/kg) the false suspect rate was 3% for the strip test and < 0.1% for the ELISA test. Finally, the false negative rate for samples contaminated at levels above the STC (75 ng/kg in the present case) resulted to be 0.4% for strip test and 0.1% for ELISA. In both cases, the acceptability criterion of maximum 5% false negative rate was met. 

The overall results indicated satisfactory kits reliability in discriminating samples contaminated at different AFM_1_ levels set in a very narrow working range (from ≤ 0.5 to 75 ng/kg), encompassing EU regulatory limits. 

In addition, the method fitness for purpose of evaluating milk contamination at the alert threshold of 40 ng/kg was evaluated by analyzing 20 contaminated samples from two different farms. The obtained average responses were 40.0 ng/kg for the strip test and 40.2 for the ELISA, with relative standard deviation (RSD_ip_) of 9.8% and 5.8%, respectively. The resulting cut-off levels were 33.2 ng/kg and 36.1 ng/kg. No false suspect samples resulted for blanks with respect to these cut-off values. These data demonstrated the fitness for purpose of the two tested kits in evaluating compliance of milk samples with respect to the alert threshold of 40 ng/kg.

### 2.2. Verification of Method Performances through Quality Control Data 

Verification of method performances was carried out through long-term intra-laboratory QC measurements over a period of 12 months (see Section 4.6). The results of the validation and the verification study were compared in terms of precision, recovery rates and cut-off values, as shown in Table 2. The cut-off values calculated by QC data matched very well with those obtained by validation data. Moreover, the data from the validation study as well as from the QC exercise revealed comparable values for the precision and the recovery rate, thus demonstrating sufficient ruggedness of both methods over the time and different production lots. 

### 2.3. Analysis of Naturally Contaminated Samples

The trueness of data generated by the two screening methods was evaluated by comparing them with results obtained by the reference AOAC Official Method 2000.08 on a set of raw cow milk samples naturally contaminated in the range n.d. (≤0.5 ng/kg) – 50 ng/kg AFM_1_. 

**Figure 1:**
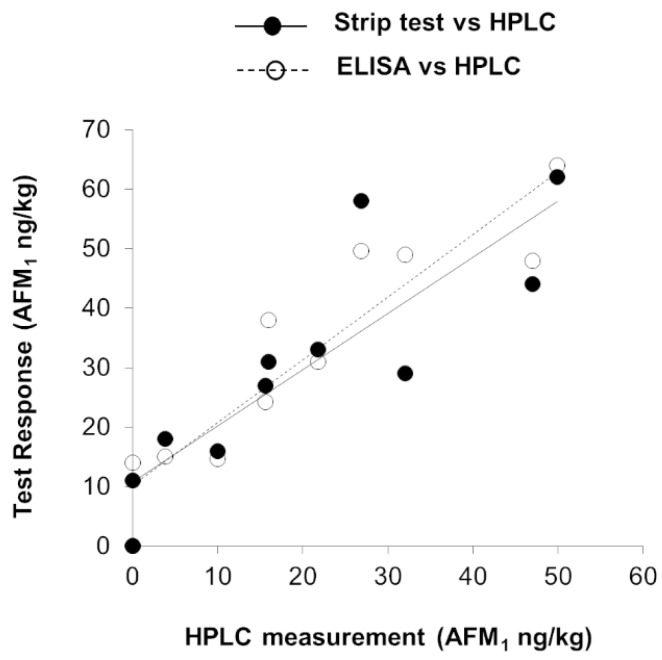
Correlation between results (AFM_1_ mass fraction, ng/kg) obtained by strip test or ELISA and the HPLC analysis performed according to the AOAC Official Method 2000.08.

Results are depicted in Figure 1. The two test kits performed in a similar way, and in both cases a satisfactory correlation was observed, with results provided by the reference method (*r* = 0.923 and slope = 0.84 for strip test vs HPLC and *r* = 0.924 and slope = 1.05 for ELISA vs HPLC). Irrespective of a slight overestimation of the AFM_1_ content in some of the blank samples (HPLC result ≤ 0.5 ng/kg), both immunoassays returned values lower than 14 ng/kg, confirming the absence of false suspect results.

### 2.4. Extension of Scope of the Method to Other Commodities

Finally, the extension of the scope of the strip test method to goat and sheep milk was evaluated by applying the experimental design foreseen in the EU regulation. The regulation foresees that “as long as the new commodity belongs to a commodity group (“milk” in the present case) for which an initial validation has already been performed, a minimum of 10 homogeneous negative control and 10 homogeneous positive control (at STC) samples shall be analyzed under intermediate precision conditions. The positive control samples shall all be above the cut-off value as calculated in validation experiments.

**Figure 2:**
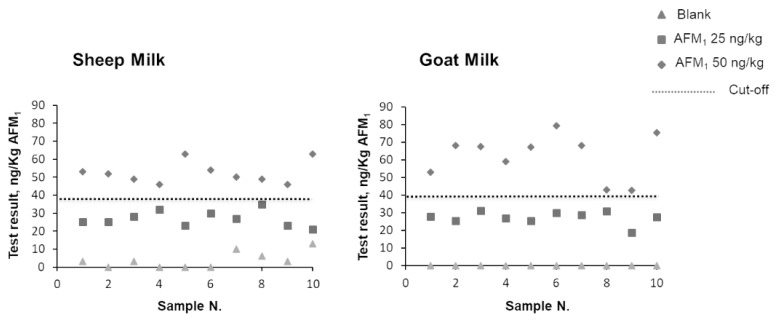
Results of strip test analysis of blank sheep and goat milk samples and samples contaminated with 25 and 50 ng/kg AFM_1_.

For these purposes, first specific calibration curves (bar codes) were generated for strip test analysis of raw goat and raw sheep milk. Then 10 blank (negative) samples and 10 samples contaminated by AFM_1_ at 50 ng/kg were analyzed for each milk type. An additional sample set containing AFM_1_ at 25 ng/kg was also included. Results are reported in Figure 2. In both cases negative samples were correctly classified as below the cut-off. No false suspect was reported. In addition, samples contaminated at 50% STC (25 ng/kg) were all correctly classified as below the cut-off (Table 1) and no false suspect was reported. All samples contaminated at 50 ng/kg (STC) were correctly classified above the cut-off. 

The obtained data showed the applicability of the strip test immunoassay to goat and sheep milk provided that a specific calibration curve was used.

### 2.5. Fitness for Purpose of the Validated Immunoassays

Validation experiments returned, for both immunoassays, fit for purpose analytical performances such as cut-off values (37.7 ng/kg and 47.5 ng/kg for strip test and ELISA respectively), false suspect rate for blanks (<0.1% for both assays) and false negative rate (<0.4% (for both assays). Both assays showed an intermediate precision at STC (50 ng/kg) <17% either in validation and QC measurements. However, besides analytical performances, when choosing a method for rapid mycotoxin screening, the concept of fitness for purpose also includes some practical parameters. Factors such as the time needed for analysis, the skills or level of education of the user of the method and the place where the analysis needs to be carried out are generally taken into consideration by the end users. A more comprehensive comparison of performances of mycotoxin screening tests can be found in Lattanzio et al. [29]. In the present case, the total analytical time for strip test assay was about 10 min and the use of the incubator, as well as the portable reader, made it suitable for on farm use. The ELISA involved more steps, a basic laboratory equipment and more time (approx 80 min). On the other hand, ELISA tests allow to handle up to 48 samples simultaneously (including calibrants and QC samples), while the strip test foresees only one sample per analysis/strip. ELISA can be therefore more efficient when a large number of (sub)samples need to be analyzed in a short period of time. On the other hand, when applied in routine by experienced technicians, strip testing can be stacked to process multiple samples in a relatively short period of time, by processing 10 to 15 samples 1 min apart. Finally, concerning method transferability to unskilled personnel, the strip test appears easier to be applied by low experienced technicians, not only because the analytical protocol is less laborious, but also because the automatic calibration via QR code uploading. In principle both platforms are potentially suitable for multiplexing [30,31,32].

## 3. Conclusions

Analytical performances and fitness for purpose of two commercial immunoassays widely applied for the detection of AFM_1_ in milk (strip test and ELISA) were evaluated, according to guidelines set in Regulation 519/2014/EU. Both assays showed satisfactory performances in terms of precision, recovery rates, false positive and false negative rates. In addition, the method performance profiles of the two methods obtained in the validation study could be verified by long-term intra-laboratory QC data. A good correlation between the results provided by the validated assays and the AOAC reference method was observed when analyzing naturally contaminated samples. The extension of the scope of the strip test method to goat and sheep milk was successfully evaluated by applying the experimental design foreseen in the EU regulation. 

## 4. Materials and Methods 

### 4.1. Materials

Methanol and acetonitrile (HPLC grade) were purchased from Mallinckrodt Baker (Milan, Italy). Ultrapure water was produced by using a Millipore Milli-Q system (Millipore, Bedford, MA, USA). AflaM1 Test immunoaffinity columns were from VICAM (A Waters Business, Milford, MA, USA). Paper Filters (Whatman 4) were obtained from Whatman International Ltd. (Maidstone, UK). Standard aflatoxin M_1_ (acetonitrile solution 10 µg/mL) was purchased from Supelco (Bellefonte, PA, USA).

### 4.2. Milk Samples

Milk samples (cow, sheep, goat) were collected from farm bulk tanks from Italian farmers in the time span 2018–2019. After collection samples were stored at 4 °C and analyzed within 48 h.

Fortified milk samples to be used for validation (Section 4.6) were prepared as follows. A spiking solution containing AFM_1_ at 1 µg/mL was prepared by diluting 10 times the standard AFM_1_ solution. Then 25 µL of spiking solution were added to 50 mL of milk to prepare a mother solution at 500 ng/kg AFM_1_. The mother solution was diluted by appropriate volumes of milk to obtain contaminated samples at 75, 50, 40 and 25 ng/kg.

### 4.3. Strip Test Immunoassay

The strip test (AFLAM1-V™), incubator, and a photometric reader (Vertu Reader) were from VICAM (A Waters Business, Milford, MA, USA). The strip test format is based on an indirect competitive immunoassay. Line intensities developed on the strip membrane (test line and control line) are measured using the photometric reader. The test response is the ratio between the signal intensity of the test line and that of the control line and it is converted into toxin concentration through a lot specific calibration curve.

Strip test analyses of milk samples were performed as follows. Two hundred microliters of cold milk were pipetted into the reagent vial. After vortex mixing (3 times x 5 sec) the vial was placed into the incubator set at 40 °C and the strip was inserted into it. The sample was allowed to migrate onto the strip for to 10 min, then the strip was placed into the reader holder for result reading. The lot specific calibration curve was uploaded onto the reader system by using the corresponding barcode provided by the supplier. The calibration curve was generated by spiking uncontaminated milk (cow, sheep or goat milk) at seven AFM_1_ levels over the range 0–800 ng/kg, performing triplicate measurements for each calibration level. The limit of detection declared by the supplier was 25 ng/kg.

### 4.4. Enzyme Linked Immunosorbent Assay

The ELISA kit (I’Screen AflaM1) was from Eurofins Technologies (Budapest, Hungary). Calibration standards, conjugate, antibody and substrate/chromogen solutions were provided in the kit. The plate reader was Multiskan MS Plus MK II ELISA reader from Labsystems (Helsinki, Finland).

Samples were analyzed as follows. One hundred microliters of milk (or calibrant solution) were transferred into the well and incubated for 45 min at room temperature. After discarding the liquid by turning the plate upside down, the wells were filled completely with the working wash solution. Then the liquid was poured out from wells and the remaining drops were removed by tapping the microplate upside down against adsorbent paper. This washing sequence was repeated four times. Then 100 µL of AFM_1_-enzyme conjugate solution were added and incubated for 15 min. After discarding the liquid, the wells were washed four times, according to the above described procedure. Then, 100 µL of substrate solution were added and incubated for 15 min for color development. Finally, 50 µL of stop solution were added. Result were read measuring the absorbance at 450 nm. The limit of detection declared by the supplier was 5 ng/kg.

### 4.5. Reference Method (AOAC Official Method 2000.08)

Screening for blank samples to be used for validation experiments and strip test calibration curve generation, and analysis of naturally contaminated samples for method comparison purposes were performed, according to the AOAC Official Method 2000.08, with minor modifications. Briefly, milk samples (50 mL) were centrifuged at 2000× *g* to separate the fat. After discarding the upper thin fat layer, the sample was filtered through paper filter. The filtered sample (25 mL) was passed through the immunoaffinity column. The eluate was discarded and the column was washed twice with 10 mL distilled water. The toxin was eluted by 2 × 1 mL methanol. The eluate was collected and dried down under nitrogen stream. The residue was re-dissolved with 250 µL of a mixture water:acetonitrile (75:25 by vol).

HPLC-FD analyses were performed on an Agilent 1100 Series chromatographic system (Agilent Technologies, Palo Alto, CA, USA) equipped with a fluorometric detector (model 363), and the ChemStation data software (Agilent Technologies). The analytical column was a Zorbax SB-C18 Rapid Resolution HT (4.6 mm × 50 mm, 1.8 µm) with corresponding in-line filter. The chromatographic separation was performed by a gradient elution using water (solvent A) and acetonitrile (solvent B). The flow rate of the mobile phase was 1 mL/min. The injection volume was 50 µL. The column was kept at a temperature of 40 °C; the excitation wavelength was set at 365 nm and the emission wavelength was set at 435 nm. The detection limit was 0.5 ng/kg.

### 4.6. Validation Design and Verification Study via Qualitity Control (QC) Measurements

The single laboratory validation study was designed to fulfil the specifications established in Commision Regulation (EU) 519/2014, in terms of minimum sample set and minimum number of validation levels.

Measurements were distributed in two different days (instead of 5 as suggested in the regulation, due to the limited stability of milk samples). Milk samples were from three different farms. In addition, each sample was analyzed in quadruplicate each day under repeatability conditions. The design resulted in 12 independent analysis per day and 24 measurements in total per each validation level.

The screening target concentration (STC) was set at the EU maximum permitted level of 50 ng/kg. The selected validation levels were four: blank (i.e., <0.5 ng/kg), and samples spiked by 25 ng/kg (50% STC), 50 ng/kg (STC), 75 ng/kg (150% STC) of AFM_1_. An additional sample set containing AFM_1_ at 40 ng/kg was included to evaluate the fitness for purpose of the two tested methods in high risk periods when it is recommended to set the alert threshold (STC) at 40 ng/kg [18].

The results of analysis were then subjected to statistical assessment to calculate validation parameters as described in the following. 

Verification of method performances was carried out through long-term intra-laboratory QC measurements. For both assays, 50 measurements of the QC material, i.e., raw cow milk spiked at STC (50 ng/kg), were spread over a period of 12 months. Moreover, 4 different kit lots were used for the strip test and 6 different lots for the ELISA test, thus including additional factors in the verification study, which may have an impact on the result of analysis. Finally, the results of these analysis were taken as a basis for the calculation of the cut-off values, precision and recovery rates. 

#### 4.6.1. Precision

To evaluate the precision profile of the method, data generated each validation level were subjected to analysis of variance (ANOVA). When applying this model, as defined by ISO 5725 [33], the measured test response Y_ijk_ (the AFM_1_ mass fraction in the present case) is defined as the true value (TV) plus the contribution of 3 components:

Y_ijk_ = TV + D_i_ + M_ij_ +R_ijk_ (1)

where D_i_ is the between-day variability, M_ij_ is the between-matrix (milk batches from different farms) variability, and R_ijk_ is the within-day variability. The within-day variability gives the precision under repeatability conditions, whereas the sum of all components gives the intermediate precision. The statistical assessment was done with the software package MINITAB^TM^ Statistical Software for Windows (Version 15).

#### 4.6.2. Cut-Off Value

The measured levels (ng/kg) of samples containing AFM_1_ at STC were taken as basis for the calculation of the cut-off value. According to Regulation 519/2014/EU the following equation was used:

(2) $$Cut\;off=R_{STC}-tvalue_{\left(0.05\right)}\times SD_{STC}$$

where the *R_STC_* is the mean level of AFM_1_ (ng/kg) calculated from all 24 experiments performed on samples containing AFM_1_ at STC, *SD_STC_* is the corresponding standard deviation of intermediate precision as defined in the previous paragraph, and *tvalue*_(0.05)_ is the one tailed t value for a rate of false negative results of 5%.

#### 4.6.3. False Suspect and False Negative Rate

Using the cut-off value and the results from the analysis of negative samples the rate of false suspect results was estimated by first calculating the t-value as follows:

(3) $$tvalue=\frac{\left(cut\;off-mean_{neg}\right)}{SD_{neg}}$$

where *mean_neg_* is the mean value of the results obtained from the 24 experiments on the negative samples and *SD_neg_* is the corresponding standard deviation of intermediate precision. 

From the obtained t-value, based on the degrees of freedom calculated from the number of experiments (23 in the present case), the false suspect rate results (probability) for a one tailed distribution was calculated using the spread sheet function “TDIST” from Microsoft Excel. 

The false suspect rate for samples containing AFM_1_ at 50% STC was calculated by applying the same procedure using the mean value of the results obtained from the 25 experiments on samples containing AFM_1_ at 50% STC and the relevant standard deviation of intermediate precision.

Finally, the false negative rate for samples containing AFM_1_ at levels above the STC was estimated by calculating the t value as specified here:

(4) $$tvalue=\frac{\left(mean_{>STC}-cutoff\right)}{SD_{>STC}}$$

where *mean_>STC_* is the mean value of the results obtained from the experiments on the samples containing the analyte above the STC, cut-off is the value established as above, and *SD_>STC_* is the corresponding standard deviation of the intermediate precision. The probability corresponding to the calculated t value with a one-tailed distribution gives the rate of false negative results for the samples containing the analyte at levels higher than STC.

## Figures and Tables

**Table 1:** Analytical performances of the strip test immunoassay and enzyme linked immunosorbent assay (ELISA), as resulted by validation experiments.

Test Sample		Strip Test	ELISA
**Blank** (AFM_1_ ≤ 0.5 ng/kg)	Mean response (ng/kg)	4.2	8.2
	RSD_r_ (%)^1^	93	16
	RSD_ip_ (%)^2^	140	33
	False suspect rate (%)	<0.1	<0.1
**50% STC** (25 ng/kg)	Mean response (ng/kg)	23.2	30.7
	RSD_r_ (%)	26	5
	RSD_ip_ (%)	32	5
	False suspect rate (%)	3	<0.1
**STC** (50 ng/kg)	Mean response (ng/kg)	53.1	57.9
	RSD_r_ (%)	12	5
	RSD_ip_ (%)	17	10
**150% STC** (75 ng/kg)	Mean response (ng/kg)	85.6	105
	RSD_r_ (%)	16	15
	RSD_ip_ (%)	19	15
	False negative rate (%)	0.4	0.1
**Cut-off value** (ng/kg)		37.7	47.5

^1^ RSD_r_ relative standard deviation of the repeatability; ^2^ RSD_ip_ relative standard deviation for intermediate precision.

**Table 2:** Verification of strip test and ELISA method performances though quality control (QC) data.

Method Performances		Strip Test		ELISA	
		Single Lab validation	QC	Single Lab validation	QC
**STC** (50 ng/kg)	Mean response (ng/kg)	53.1	53.5	57.9	52.8
	Relative recovery rate (%)	106	107	116	106
	RSD_ip_ (%)^1^	17	12	10	9.2
	Cut-off value (ng/kg)	37.7	42.7	47.5	44.7

^1^ RSD_ip_ relative standard deviation for intermediate precision.
